# Expression of methionine adenosyltransferase 2A in renal cell carcinomas and potential mechanism for kidney carcinogenesis

**DOI:** 10.1186/1471-2407-14-196

**Published:** 2014-03-17

**Authors:** Xuliang Wang, Xiaoqiang Guo, Wenshui Yu, Cailing Li, Yaoting Gui, Zhiming Cai

**Affiliations:** 1 Shenzhen Key Laboratory of Genitourinary Tumor, Shenzhen Second People’s Hospital, First Affiliated Hospital of Shenzhen University, Shenzhen 518035, Guangdong, China; 2 Department of Urology, Guangdong and Shenzhen Key Laboratory of Male Reproductive Medicine and Genetics, Peking University Shenzhen Hospital, Shenzhen PKU-HKUST Medical Center, Shenzhen 518036, Guangdong, China; 3 Medical College, Shantou University, Shantou 515041, Guangdong, China

**Keywords:** Methionine adenosyltransferase 2A, Renal cell carcinomas, S-adenosylmethionine, Heme oxygenase-1

## Abstract

**Background:**

Methionine adenosyltransferase 2A (MAT2A) is an enzyme that catalyzes the formation of S-adenosylmethionine (SAMe) by joining methionine and ATP. SAMe is a methyl donor for transmethylation and has an important role for DNA and/or protein methylation. MAT2A is expressed widely in many tissues especially in kidney. Several studies have demonstrated that there are abnormal expressions of MAT2A in several kinds of cancers such as liver and colon cancers. But the relationship of MAT2A between renal cell carcinomas (RCC) is less understood.

**Methods:**

The mRNA expression level of the *MAT2A* gene was determined in 24 RCC patients and 4 RCC cell lines, using real-time quantitative-polymerase chain reaction (RT-PCR). The MAT2A protein content was measured by western blotting and immunohistochemical analysis in 55 RCC patients. The mRNA levels of heme oxygenase-1 (HO-1) and cyclooxygenase-2 (COX-2) were also analysized in patients using RT-PCR. The correlations between the MAT2A and HO-1 as well as COX-2 were analyzed with nonparametric Spearman method.

**Results:**

MAT2A transcript was significantly downregulated in cancer tissues compared to normal tissues (P < 0.05). Immunohistochemical analysis and western blotting indicated that level of MAT2A protein was decreased in cancer tissues. The statistical analysis reveals a negative correlation between MAT2A and HO-1 expression in RCC patients and cell lines (P < 0.01).

**Conclusions:**

This study demonstrated that MAT2A was lower expression in cancer tissues, suggesting that it may be involved in the development of RCC. MAT2A is a transcriptional corepressor for HO-1 expression by supplying SAM for methyltransferases, which may be one of potential mechanism of MAT2A as tumor suppressor in kidney carcinogenesis.

## Background

Kidney cancer is among the 10 most common cancers, which accounts for 2% to 3% of all adult malignancies and causes 100,000 deaths per year worldwide [1]. The most common hisitologic subtype of kidney cancer is renal cell carcinomas (RCC), of which 70–80% of cases are defined as clear cell renal cell carcinoma (ccRCC) [2]. RCC is generally resistant to chemotherapy and radiation therapy [3]. Radical or partial nephrectomy of the tumor at an early stage remains the mainstay of curative therapy nevertheless up to 40% of the patients relapse after surgery [4]. Unlike other solid malignancies, methods for RCC early diagnosis are lacking but they are critically important because therapeutic efficacy and, hence, survival are tightly linked to the time of diagnosis. Distant metastases are present at the time of initial diagnosis in approximately one third of patients, and the tumor will recur in another third, even after nephrectomy with curative intent [5]. Better understanding of the molecular mechanisms of RCC may hasten identification of new prognostic markers and development of new diagnostic and therapeutic strategies.

Cancer cell metabolism is significantly altered compared with metabolism of normal cells. Significant progresses on genetics of renal cancer have proved that it is a metabolic disease [6]. Several known genes related kidney cancer, such as von Hippel-Lindau (VHL), fumarate hydratase (FH) and succinate dehydrogenase (SDH) are involved in pathways that respond to metabolic stress [7]. VHL loss can increase the expression of hypoxia-inducible factors, which affect several metabolic pathways, including glycolysis and oxidative phosphorylation [8]. The mutations of FH and SDH are associated with dysfunction of tricarboxylic acid cycle [9,10]. So, it will provide the foundation for the development of effective therapy for kidney cancer to understand the metabolic basis of this disease [11].

One-carbon metabolism can integrate nutritional status from amino acids, glucose and vitamins, which is important for the biosynthesis of lipids, nucleotides and proteins, the maintenance of redox status and the substrates for methylation reactions [12]. One-carbon metabolism involves in the folate and methionine cycles. The related enzymes involved folate metabolism have been discovered to be associated to RCC risk [13,14]. The abnormality of methionine cycle was identified in many kinds of cancers [15,16]. But, the relationship between methionine metabolism and RCC is poorly understood. Methionine adenosyltransferase (MAT) is an essential cellular enzyme that catalyzes the formation of S-adenosylmethionine (SAMe), the principal biological methyl donor [17]. In mammals, this essential enzyme is the product of two different genes, MAT1A and MAT2A, which display a distinct pattern of expression among different tissues. MAT1A is the predominant enzyme in liver parenchymal cells, while MAT2A is expressed in all other tissues [18].

However, the relationship between the expression of MAT2A and RCC development is still unknown. In this study, we investigated expression levels of MAT2A gene and protein in RCC specimen and cell lines. Then, we also determined the association between MAT2A expression and other RCC related genes’ expressions to understand the potential mechanism underlying MAT2A involved in RCC carcinogenesis. Our results suggest that MAT2A is downregulated in cancer tissues of RCC patients and has function of tumor suppressor though repressing the expression of heme oxygenase-1 (HO-1).

## Methods

### Patients and tissue specimens

A total of 55 paired ccRCC cancer tissues and adjacent normal tissues samples were obtained from the Biobank of Complex Diseases in Shenzhen between 2010 and 2012 in China. The adjacent normal tissues were defined as kidney tissues located 2.0 cm outside of visible ccRCC lesions. All the 55 patients’ survival information was received by telephone. The median follow-up period was 69 months (range: 4 ~ 116 months). Patients’ clinical characteristics (gender, age, size, nodal status, metastasis and Fuhrman Nuclear Grade) were obtained from the medical records (Table 1). No any treatment (chemotherapy or radiation) was used before the operation.

**Table 1:** Summary of the clinical characteristics of 55 RCC patients

**Clinical Features**		**Number of patients**
Age	Mean	60
	Range	28-79
Gender	Male	30
	Female	25
TMN Stage	T1a	20
	T1b	16
	T2	12
	T3/T4	7
Size (cm)	Mean	4.3
	Range	1.3-11
Location	Side (Left/Right)	25/30
	Upper pole	30
	Middle pole	18
	Lower pole	7
Fuhrman Grade	Grade 1	28
	Grade 2	20
	Grade 3	4
	Grade 4	3
Lymph node	Negative	52
	Positive	3

All resection samples were confirmed to be ccRCC by clinical pathology and carbonic anhydrase 9 (CA-9) measurements (Additional file 1: Figure S1). The collection and use of the patient samples were reviewed and approved by Institutional Ethics Committees of Peking University Shenzhen Hospital, and written informed consent from all patients was appropriately obtained. Frozen tissues from 24 ccRCC cancer and adjacent normal samples were randomly selected from all 55 paired samples for extraction of total RNA.

### Cell culture

The human renal cancer cell lines (ACHN, Caki-1,769-P and 786-O) and embryonic kidney cell (HEK293) were obtained from cell resource center of Shanghai Institutes for Biological Sciences, Chinese Academy of Science. All cells were maintained in Dulbecco’s modified Eagle’s medium (GIBCO, Grand Island, USA) supplemented with 10% fetal bovine serum (FBS, Hyclone, Logan, USA), 50 U/mL penicillin and 50 μg/mL streptomycin. Cells were grown in a humidified atmosphere with 5% CO_2_ at 37°C. Cells were collected for following study.

### RNA extraction and cDNA synthesis

Total RNA was extracted from cancer tissues, normal adjacent tissues and 5 cells with Trizol reagent (Invitrogen, Carlsbad, CA, USA) according to the manufacturer’s protocol. The concentration of total RNA was determined using a NanoDrop ND-1000 spectrophotometer (Thermo Scientific, Wilmington, DE, USA). Then, cDNA was synthesized from 1 μg of total RNA using a Fermentas RT system (Thermo Scientific, Wilmington, DE, USA), according to the manufacturer’s instructions. Reverse transcription reactions were carried out at 25°C for 5 mins and followed by 42°C for 60 mins.

### Quantitative real-time polymerase chain reaction (qRT-PCR)

The mRNA expression levels were analyzed using SYBR Premix Ex TaqTM II (Takara, Dalian, China), with β-Actin as an internal reference. qRT-PCR was performed in 20 μl reaction mixture containing 10 μl of SYBR Premix, 0.5 μM of forward and reverse primers, and 1 μl template cDNA on LightCycler480 System (Roche, Foster City, CA, USA). The primers were designed according to the human MAT2A, CA-9, heme oxygenase-1(HO-1), cyclooxygenase-2 (COX-2) and β-Actin genes sequences reported in GenBank. The primer sequences were synthesized by Invitrogen (Guangzhou, China) as follows:

MAT2A, Forward primer: 5′-ATGAACGGACAGCTCAACGG-3′,

Reverse primer: 5′-CCAGCAAGAAGGATCATTCCAG-3′;

CA-9, Forward primer: 5′- GGATCTACCTACTGTTGAGGCT-3′,

Reverse primer: 5′- CATAGCGCCAATGACTCTGGT-3′;

HO-1, Forward primer: 5′-ATGACACCAAGGACCAGAGC -3′,

Reverse primer: 5′-GTGTAAGGACCCATCGGAGA -3′;

COX-2, Forward primer: 5′-CTGGCGCTCAGCCATACAG-3′,

Reverse primer: 5′-CGCACTTATACTGGTCAAATCCC-3′;

β-Actin, Forward primer: 5′- CCACTGGCATCGTGATGGACTCC -3′,

Reverse primer: 5′-GCCGTGGTGGTGAAGCTGTAGC-3′;

All reactions were incubated at 95°C for 5 min, followed by 40 cycles of 95°C for 10 s, 60°C for 20 s and 72°C for 30 s. PCR reactions of each sample were conducted in duplicate. Data were analyzed through the comparative threshold cycle (CT) method.

### Western blotting

Five cells, cancer tissues and adjacent normal tissues from all patients were homogenized in radioimmunoprecipitation assay buffer (RIPA) containing the protease inhibitors phenylmethylsulfonyl fluoride (100 μg/mL), cocktail (1 mmol/L) and dithiothreitol (0.5 mmol/L). Homogenates were centrifuged and supernatants were collected. Protein concentrations were determined by bicinchoninic acid (BCA) protein assay kit (Thermo Pierce ). A total of 50 μg of protein from each sample was resolved by reducing loading buffer and separated by 10% sodiumdodecyl sulfate-polyacrylamide gel electrophoresis (SDS-PAGE) followed by electrophoretic transfer to a polyvinylidene difluoride (PVDF) membrane. The PVDF membrane was saturated with 5% skim milk in TBST (50 mM Tris–HCl, 150 mM NaCl, 0.1% Tween-20) for 2 h and then incubated with primary antibodies at 4°C overnight. The primary antibodies used included rabbit polyclonal antibodies to MAT2A (1:1000, Abcam, Hong Kong, China), HO-1(1:200, Santa Cruz, Shanghai, China) and β-actin (1:5,000, Abcam, Hong Kong, China). The specificity of the MAT2A antibody has been determined (Additional file 2: Figure S2). PVDF membrane was incubated with 1:10,000-diluted peroxidase-coupled goat anti-rabbit immunoglobulin G (IgG) (secondary antibody, EarthOx, San Francisco, USA) for 1 h, after washing three times with TBST (5 min/time) at room temperature. After further washing with TBST four times, the PVDF membrane was exposed to enhanced chemiluminescence substrate (Millipore, Rockford, USA) for 30 min and detection was performed using a film.

### Immunohistochemical analysis

Paraffin sections (3 μm) from samples of 55 ccRCC samples and adjacent normal samples were deparaffinized in 100% xylene and re-hydrated in descending ethanol series (100%, 90%, 80%, 70% ethanol) and water according to standard protocol. Heat-induced antigen retrieval was performed in 10 mM citrate buffer for 2 min at 100°C. Endogenous peroxidase activity and non-specific antigen were blocked with peroxidase blocking reagent containing 3% hydrogen peroxide and serum, followed by incubation with rabbit anti-human MAT2A antibody for 1 h at 37°C. After washing, the sections were incubated with biotin-labelled goat anti-rabbit antibody for 10 min at room temperature, and subsequently were incubated with streptavidin-conjugated horseradish peroxidase (HRP) (Maixin Inc, China). The peroxidase reaction was developed using 3, 3-diaminobenzidine chromogen solution in DAB buffer substrate. Sections were visualized with DAB and counterstained with hematoxylin, mounted in neutral gum, and analyzed using a bright field microscope.

All of the IHC staining results were reviewed independently by two pathologists. Positive expression of MAT2A was defined as the brown staining in the cytoplasm and nucleus. The staining results for MAT2A were semiquantitatively scored. Intensity was estimated in comparison to the control and scored as follows: 0, negative staining; 1, weak staining; 2, moderate staining; and 3, strong staining. Scores representing the percentage of tumor cells stained positive were as follows: 0, no positive cell; 1, <5%; 2, 6–25%; 3, 26–50%; 4, 51–75%; and 5, > 75%. A final score was calculated by multiplying the scores for intensity and percentage.

### Statistical analysis

Statistical analysis was carried out using the SPSS 13.0 statistical software package. qRT-PCR and immunohistochemical data were analyzed by two-tailed paired *t*-test and Mann–Whitney U test (α = 0.05). The nonparametric Spearman rank correlation coefficient was used to calculate the correlation between the MAT2A and HO-1 as well as COX-2 expressions. For all analyses, p < 0.05 was considered significant.

## Result

### Downregulated mRNA expression of MAT2A in ccRCC patients and kidney cancer cell lines

**Figure 1:**
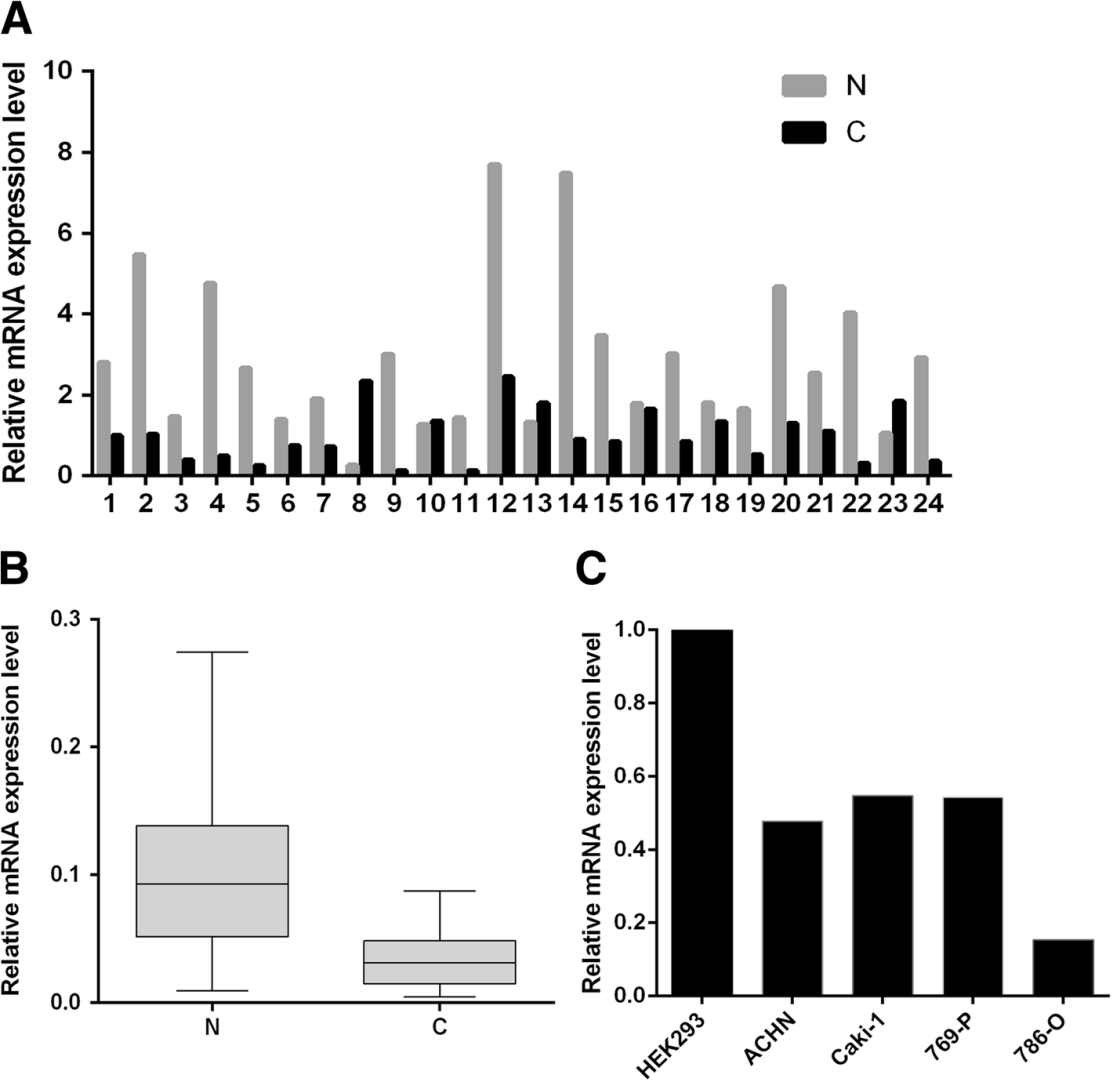
**The mRNA level analysis of the MAT2A in RCC patients and cell lines.** Total RNA of 24 RCC patients and 4 RCC cell lines was extracted and reverse transcripted to cDNA. Then, real-time qRT-PCR was carried out to determine the mRNA expression levels of MAT2A. **A**. Relative mRNA expression level of MAT2A in RCC cancer tissues and paired normal tissues of 24 RCC patients. **B**. Relative mRNA expression level of MAT2A was lower in RCC cancer tissues (C) than in paired normal tissues (N) (n = 24; P < 0.05). **C**. Relative mRNA expression level of MAT2A was lower in 4 RCC cell lines than in HEK293.

MAT2A expression in ccRCC has yet to be explored. Therefore, we first examined the transcription level of the MAT2A in cancer tissues and adjacent normal tissues from 24 RCC patients using qRT-PCR. Analysis of mRNA levels reveals 19/24 (79.2%) of RCC patients have reduced MAT2A mRNA level in cancer tissues. Moreover, 16/24 patient samples (66.7%) demonstrated a greater than twofold reduction (Figure 1A). Overall, the average reduction in MAT2A mRNA levels was 3.4 fold (P < 0.05, Figure 1B). Otherwise, the mRNA expressions in all four RCC cell lines were also downregulated relative to HEK293.

### Reduced protein content of MAT2A in ccRCC

**Figure 2:**
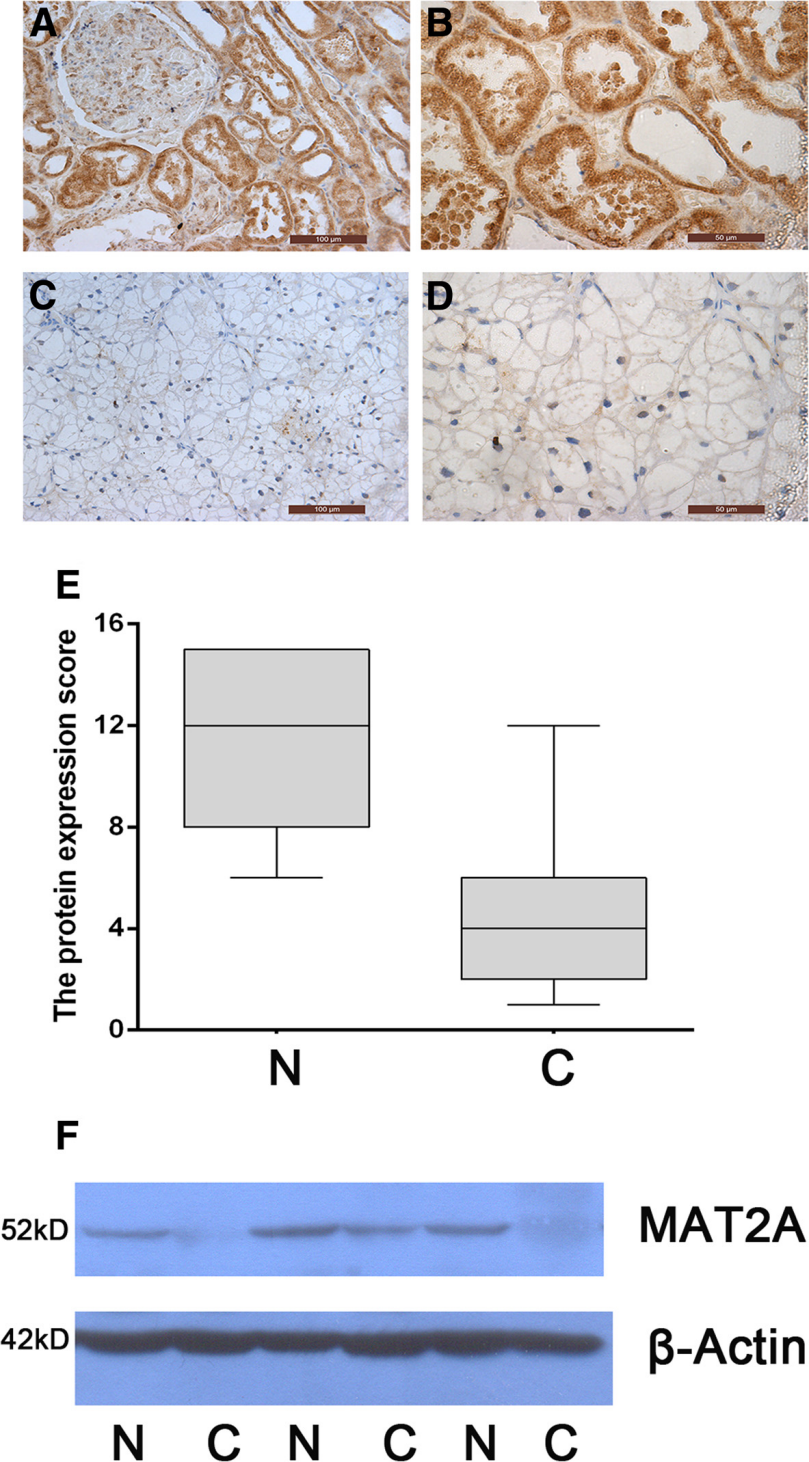
**The protein expression level of MTA2A in RCC patients. A-D** Immunohistochemical analysis of MAT2A expression. MAT2A protein content was obviously lower in cancer tissues (**C** and **D**) than in normal tissues (**A** and **B**). Magnifications × 200 (**A** and **C**) and × 400 (**B** and **D**). **E** Level of MAT2A protein was lower in RCC cancer samples (C) than in paired normal tissuesamples (N) (n = 55, P < 0.001). The MAT2A protein were semiquantitatively scored according to staining intensity and percentage in immunohistochemical analysis of cancer or adjacent tissues. **F** Western blotting analysis of MAT2A. The protein expression level of MAT2A was lower in RCC cancer tissues (C) than in paired normal tissues (N).

To support the change in mRNA level, the protein content of MAT2A was further measured by immunohistochemical and western blotting analysis. The immunohistochemical examinations indicated that MAT2A protein is mainly present in nuclei and level of it was obviously downregulated in cancer tissues compared to adjacent normal tissues (Figure 2A-D). The lower level is approximately 3.4 times (P < 0.001, Figure 2E). The western blotting analysis showed similar trend with immunohistochemical result in that protein content of MAT2A was less in cancer tissues relative to adjacent normal tissues (Figure 2F).

### Negative correlation of gene expression between MAT2A and HO-1

**Figure 3:**
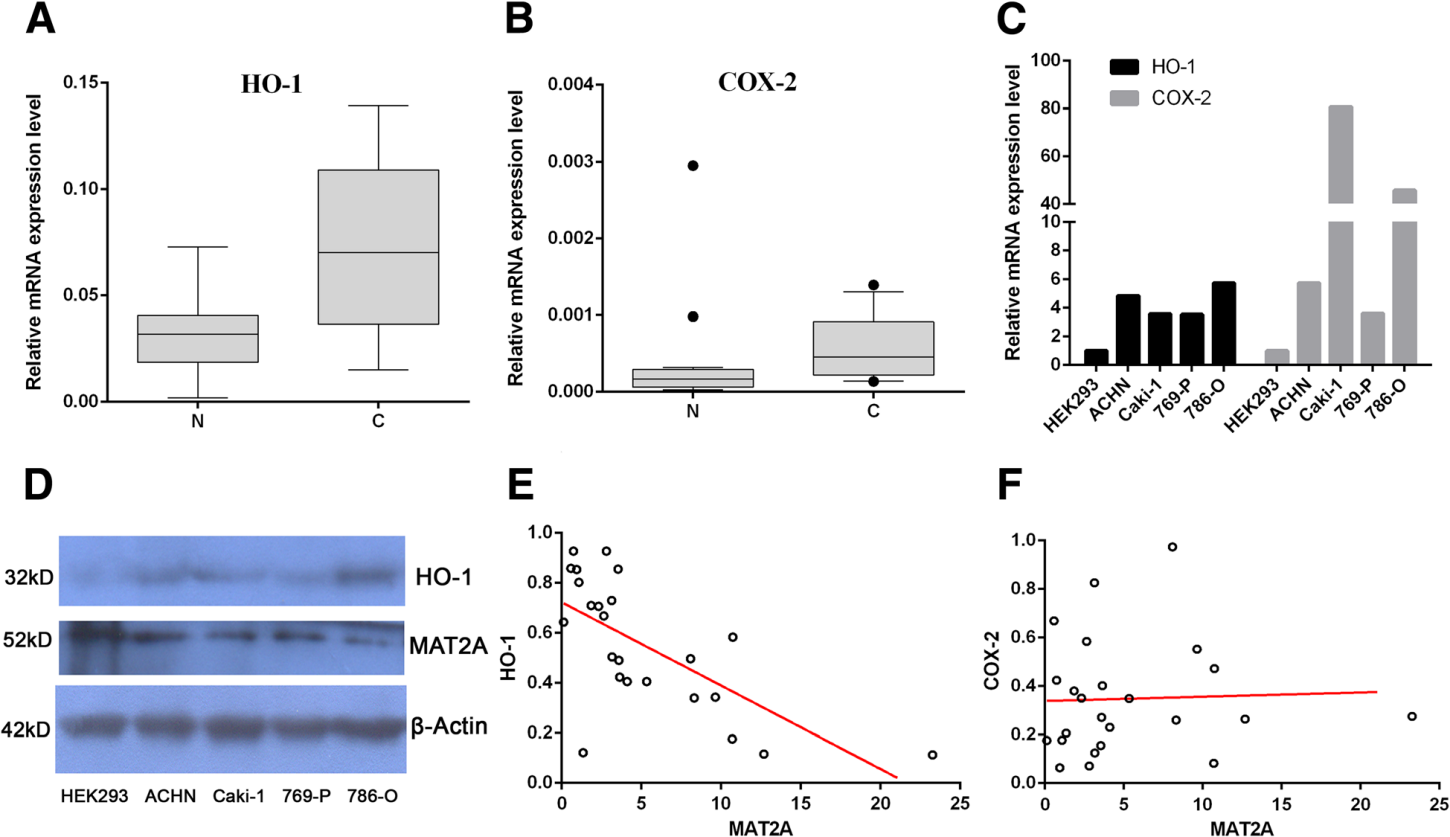
**The negative correlation between MAT2A and HO-1 expression.** mRNA levels of HO-1 and COX-2 were analyzed with real-time qRT-PCR. The correlation analysis was performed between MAT2A and HO-1 as well as COX-2 in RCC patients. **A** and **B** Relative mRNA expression levels of HO-1 **(A)** and COX-2 **(B)** were higher in RCC cancer tissues **(C)** than in paired normal tissues (N) (n = 24; P < 0.05). **C** Relative mRNA expression level of HO-1 and COX-2 were higher in 4 RCC cell lines than in HEK293. **D** The western blotting analysis of MAT2A and HO-1 in cell lines.The correlation of protein content between MAT2A and HO-1 is negative. **E** The statistical analysis reveals a negative correlation between MAT2A and HO-1 expression in RCC patients (P < 0.01). **F** The statistical analysis reveals no significant correlation between MAT2A and COX-2 expression in RCC patients.

In order to understand the potential mechanism of MAT2A, we further measure the expressions of two kidney cancer related genes *COX-2* and *HO-1* in RCC patients and cell lines. The results indicate that both genes are highly expressed in cancer tissues than in adjacent normal tissues (P < 0.01, Figures 3A and 3B).The mRNA levels are also upregulated in four RCC cell lines than in HEK293 (Figures 3C). The protein content of HO-1 is obviously higher in four RCC cell lines than in HEK293 while MAT2A shows the opposite style (Figures 3D). The statistical analysis reveals a negative correlation between MAT2A and HO-1 expression in RCC patients (P < 0.01, Figures 3E). The correlation between MAT2A and HO-1 is also negative in cell lines (Additional file 3: Figure S3). But, there is no significant correlation between MAT2A and COX-1 (Figures 3F).

## Discussion

Both DNA and histone methylation are important regulators for gene expression and chromatin structure, which have multiple effects on carcinogenesis [19,20], but the detailed mechanism is required to be determined. As a methyl donor, SAMe also plays vital role in gene expression via its effect on methylation [21]. So, MAT2A has a potential effect on tumor development and progression [22]. Recent studies have illustrated there are abnormal expressions of MAT2A in some tumors, including liver, gastric and colon cancers [23-25]. In our study, the content of MAT2A is obviously decreased in cancer tissue of RCC patients under mRNA and protein levels. So, MAT2A functions as a tumor suppressor in RCC. An increasing number of studies have suggested that MAT2A plays an important pathogenetic role in facilitating liver and colon cancer growth [26,27]. Our results further provide evidence that abnormal MAT2A is also a factor of RCC development.

Previous studies have indicated HO-1 and COX-2 are regulated by MAT2A [28]. HO-1 is an enzyme that catalyzes the degradation of heme and affords protection against programmed cell death. HO-1 is vital to fumarate hydratase deficient kidney cells survival and inhibition of it can lead to cell death [29]. It has been demonstrated HO-1 is often overexpressed in RCC patients and cell lines, and promotes survival of renal cancer cells [30,31]. COX-2 is an enzyme which catalyzes the synthesis of prostaglandins from arachidonic acid. It has been also demonstrated that COX-2 is increased in RCC and plays an important role in the proliferation of malignant renal cells [32,33]. Our results also confirmed both HO-1 and COX-2 are upregulated in RCC patients and cell lines, but further evidence indicates MAT2A is negative correlation with HO-1, no COX-2. It means that MAT2A biological role in RCC seems to be mainly associated with HO-1.

**Figure 4:**
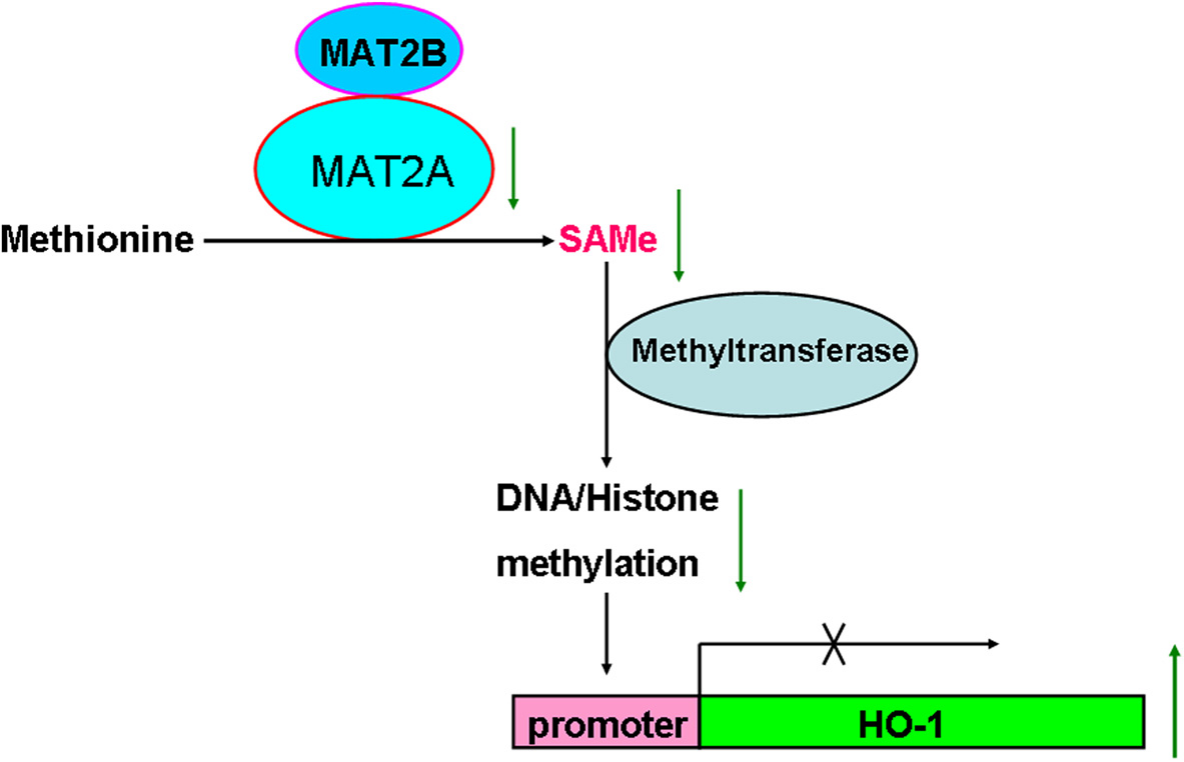
**The proposed model of MAT2A role on RCC development.** The lower content of MAT2A level reduces the product of S-adenosylmethionine (SAMe) and then decreases the level of methylation, which leads to the reactivation of HO-1 expression to increase the cell proliferation and inhibit cell apoptosis.

It has been indicated MAT2A can inhibit the expression of HO-1 as a transcriptional corepressor [28], which supplies SAMe for DNA and histone methyltransferases. MAT2A can interact with many chromatin-related proteins of diverse functions such as histone modification, chromatin remodeling, transcription regulation, and nucleo-cytoplasmic transport [34]. DNA methylation and histone modification are known to be closely related to carcinogenesis and cancer progression [35]. So, lower level of MAT2A can re-activate HO-1 to promote cell proliferation because of reducing methylation on HO-1 promoter. Accordingly, we propose the possible mechanism underlying MAT2A involved in RCC development (Figure 4).

## Conclusion

In summary, our results reveal that downregulated expression level of MAT2A is common in cancer tissues of RCC patients. The reduced MAT2A may derepress the expression of HO-1 through lowering DNA and/or histone methylation, which can be considered as potential cause of MAT2A involved RCC suppression. The results also imply that identification of other genes regulated by MAT2A during RCC development will expand our understanding of the carcinogenesis and screening strategies in RCC. Because samples in our study are limited, whether MAT2A can be as biomarker for the early diagnosis of RCC and prognostic evaluation is to be further determined. Our study only provides a possible mechanism of MAT2A biological role, so additional research is also required to determine the link between lower MAT2A levels and RCC development.

## Competing interests

The authors declare no competing financial interests exist.

## Authors’ contributions

XG, YG, ZC were responsible for experimental design, data analysis and writing of manuscript. XW, XG and CL conducted the experiments including qRT-PCR, western blotting and immunohistochemical analysis. XG and WY were responsible for collection and histological classification of clinical specimens. All authors have read and approved the final manuscript.

## Pre-publication history

The pre-publication history for this paper can be accessed here:

http://www.biomedcentral.com/1471-2407/14/196/prepub

## Supplementary Material

Additional file 1: Figure S1 The mRNA level analysis of the CA-9 in RCC patients. Relative mRNA expression level of carbonic anhydrase 9 in RCC cancer tissues and paired normal tissues of RCC patients. Click here for file

Additional file 2: Figure S2 The specificity of the MAT2A antibody. The western blotting **(A)** and immunohistochemistry **(B)** were used to determined the specificity of the MAT2A antibody. Click here for file

Additional file 3: Figure S3 The correlation between MAT2A and HO-1 expression in cell lines. The correlation between MAT2A and HO-1 mRNA **(A)** or protein **(B)** was determined. They are obviously negative (P < 0.01). Click here for file
