# Supplementary figures and images for: Expression of methionine adenosyltransferase 2A in renal cell carcinomas and potential mechanism for kidney carcinogenesis
Source: BMC Cancer. 2014 Mar 17;14:196. doi: 10.1186/1471-2407-14-196 (PMC4003826; doi:10.1186/1471-2407-14-196)

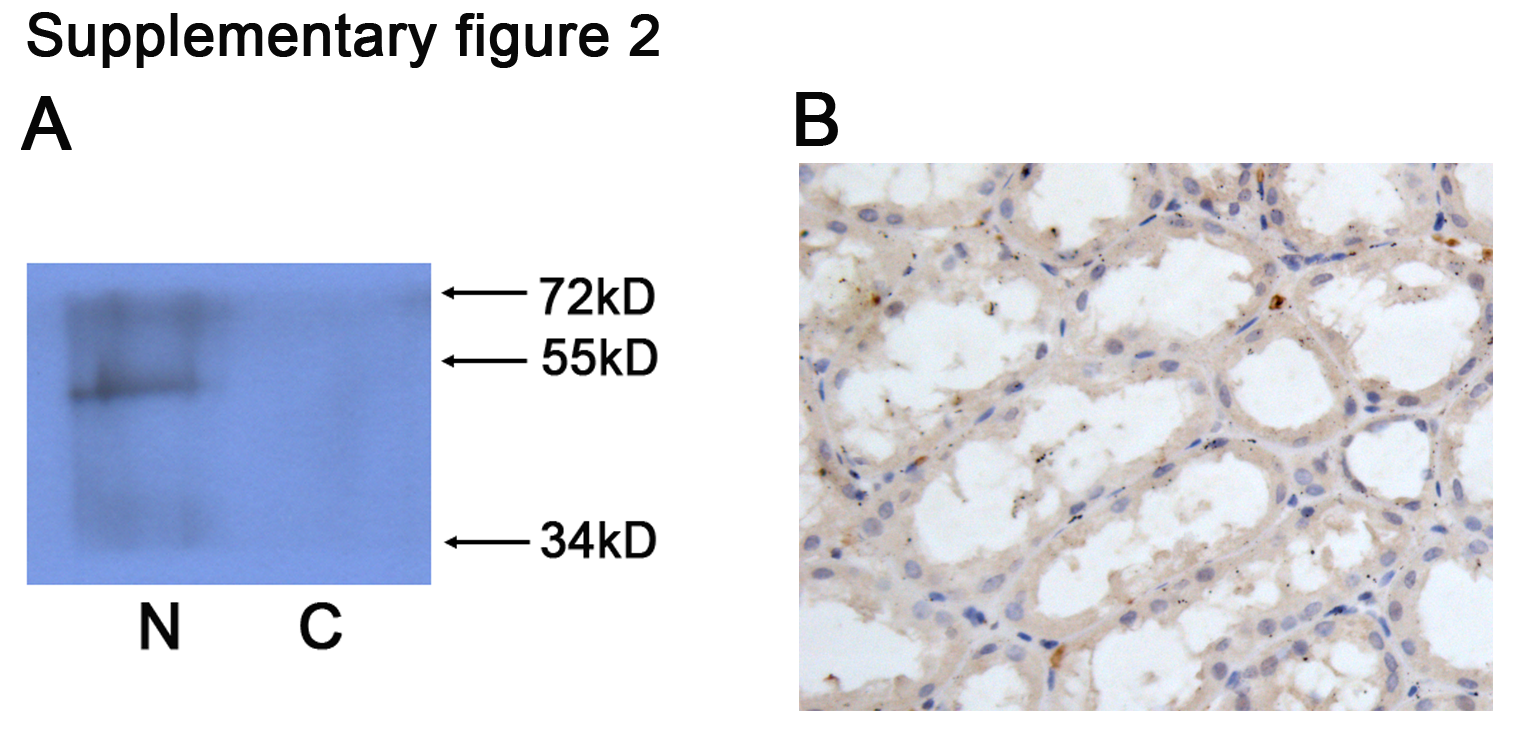

Supplement: Additional file 1: Figure S1 — The mRNA level analysis of the CA-9 in RCC patients. Relative mRNA expression level of carbonic anhydrase 9 in RCC cancer tissues and paired normal tissues of RCC patients. [file 1471-2407-14-196-S1.tiff]

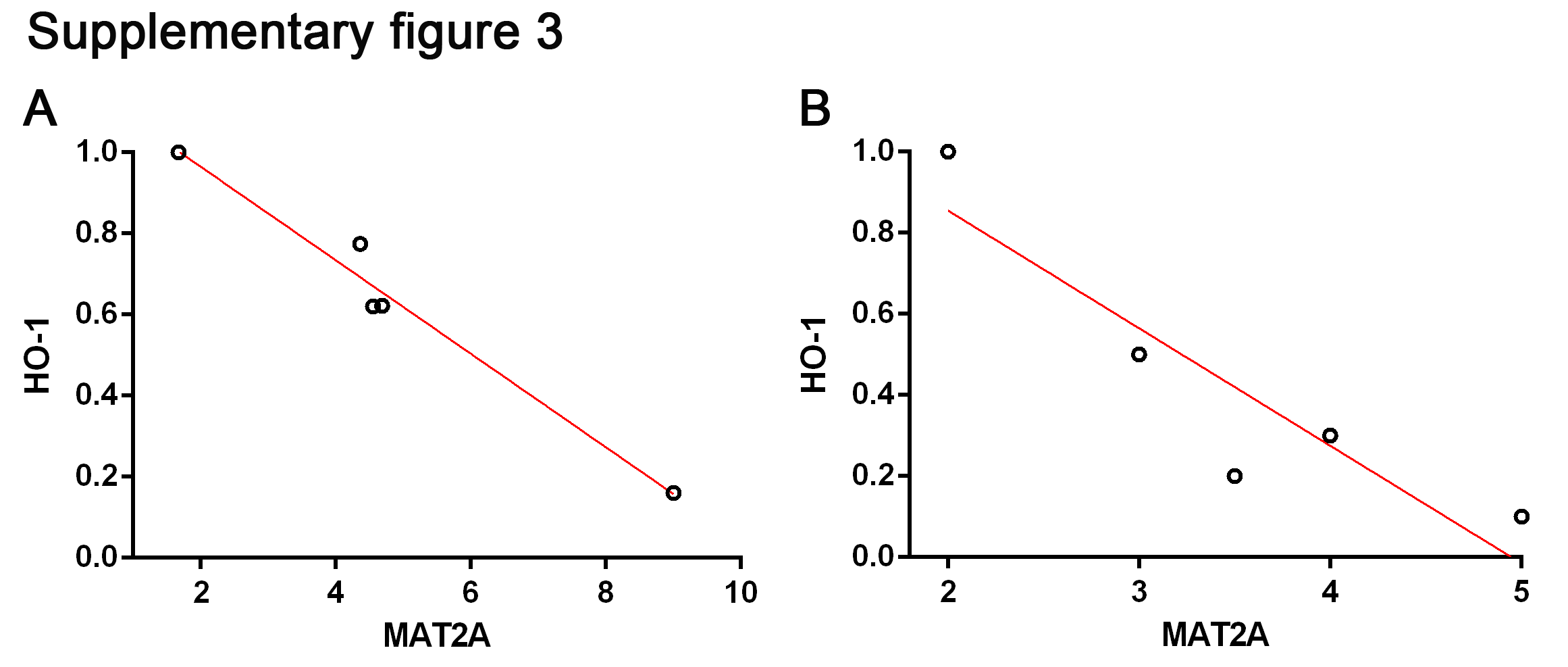

Supplement: Additional file 2: Figure S2 — The specificity of the MAT2A antibody. The western blotting (A) and immunohistochemistry (B) were used to determined the specificity of the MAT2A antibody. [file 1471-2407-14-196-S2.tiff]
